# Adipokines Vaspin and Visfatin in Obese Children

**DOI:** 10.3889/oamjms.2015.123

**Published:** 2015-11-26

**Authors:** Hassan M. Salama, Ashraf Galal, Ayat A. Motawie, Ashraf F. Kamel, Doaa M. Ibrahim, Azza A. Aly, Emman A. Hassan

**Affiliations:** 1 *National Research Centre, Pediatrics, Cairo, Egypt*; 2 *Ain Shams University, Faculty of Science, Cairo, Egypt*; 3 *National Research Centre, Department of Chemical and Clinical Pathology, Cairo, Egypt*

**Keywords:** Vaspin, visfatin, obese, children, adipokines

## Abstract

**BACKGROUND:**

Adipokines provides new insights about the physiology, pathology and treatment of obesity.

**AIM:**

We investigated the association between serum vaspin and serum visfatin concentrations with obesity in Egyptian children.

**MATERIAL AND METHODS:**

Twenty two obese children with body mass index (BMI) above 95th percentile; 11 males and 11 females were included in this study. Their mean age was 9.18 ± 2.8 years. After general clinical examination, fasting blood glucose, triglycerides, total cholesterol and high density lipoprotein cholesterol were measured in cases and controls (n=11). Fasting insulin, vaspin and visfatin were detected using ELIZA. Insulin resistance was estimated by Homeostasis model assessment method (HOMA-IR).

**RESULTS:**

Blood pressure, in both systolic and diastolic measurements was elevated significantly in obese children. Significant elevation of serum insulin and insulin resistance (HOMA/IR) were observed in obese children too. Vaspin and visfatin showed significant elevation in obese children than controls. Significant positive correlations were detected between visfatin and BMI, waist circumference, hip circumference and HOMA/IR. We found that Vaspin and visfatin are higher in obese children.

**CONCLUSION:**

Visfatin but not vaspin correlates positively with waist circumference and HOMA/IR in obese children.

## Introduction

Adipose tissue is the source of adipokines, secreted mainly by adipocytes. The rapidly growing list of adipokines provides new insights about the physiology, pathology and treatment of obesity [1]. Recently, vaspin (visceral adipose tissue-derived serpin protease inhibitor) and visfatin (also known as pre-B-cell colony-enhancing factor 1), have been identified as interesting novel adipokines having insulin-sensitizing and insulin-mimic effects, respectively [2].

Vaspin was originally identified in an animal model of obesity and type 2 diabetes. Increased vaspin mRNA expression in human adipose tissue was found to be associated with obesity [3]. Visfatin, in human, is expressed more in visceral adipose tissue than subcutaneous one. It is upregulated during inflammation [4]. Obesity and metabolic syndrome in children and adolescents is a leading cause of a low grade systemic inflammation [5].

Obesity is associated with an array of health problems in adult and pediatric populations. Adipokines are signaling to organs such as brain, liver, skeletal muscle, and the immune system—thereby modulating homeostasis, blood pressure, lipid and glucose metabolism, inflammation, and atherosclerosis [6]. The secretion of several adipokines is altered in subjects with abdominal adiposity and these changes to the endocrine balance may contribute to increased cardiovascular diseases risk [7]. The association of novel adipokines, vaspin and visfatin, with atherosclerosis is still obscure [8].

We investigated the association between serum vaspin and serum visfatin concentrations and obesity in Egyptian children.

## Subjects and Methods

### Subjects

Twenty two obese children with body mass index ((BMI) above 95^th^ percentile 11 males and 11 females were included in this study. Their mean age was 9.18 ± 2.8. Subjects are free from any other diseases. Genetic or endocrine causes of obesity were excluded from this study. Cases were not in body weight control regime or exercise at the time of the study. Eleven age and sex matched children were also included and served as controls. All controls had normal BMI ranging from 5^th^ to 85^th^ [9]. In this study BMI detected according to the Egyptian growth charts 2002 [10]. All obese and controls children underwent thorough medical examination and anthropometric measurements by one member of our team works. Informed consents were taken from the parents of all children included in this study.

### Methods

After 12 hours of fasting a blood sample was taken, and the serum was collected. Blood glucose level was determined immediately and rest of serum was stored at -80°C. Fasting blood glucose, triglycerides, total cholesterol and high density lipoprotein cholesterol were carried out using an auto analyzer (Olympus- Au-400). Low density lipoprotein cholesterol was calculated following Friedwald formula [11]. Fasting serum insulin was estimated by ELISA technique using monobind. Inc, Lack Forest, CA (92630) USA.PR. CODE: 2425.300A. Insulin resistance estimate by Homeostasis model assessment method [HOMA-IR] = [Fasting insulin (µu/ml) × Fasting glucose (mmol/litre) / 22.5 [12]. Fasting serum visfatin was assessed using ELISA using kits from CUSABIO BIOTECH CO., LTD. Catalogue No.CSB-EO8940h. Vaspin was assessed also by ELISA technique, using kit Human soluble Cluster of differentiation 100 (sCD100) ELISA kit from CUSABIO BIOTECH CO., LTD.

### Statistical Analysis

Mann-Whitney test was used for not normally distributed data and Student’s *t* tests were used for normally distributed data. Both tests are performed using the statistical version 10 programs (Stat Software Inc., Tulsa, OK, USA). The relative strength of correlations was calculated using the Spearman rank correlation coefficient (*r*_s_).

## Results

Table 1 showed the descriptive data and anthropometric measurements of cases and control group. As expected, significant differences were detected between cases and controls concerning the anthropometric measurements related to obesity. Systolic and diastolic blood pressures were significantly elevated in obese children.

**Table 1:** Demographic and Clinical Data of Obese and Control Children.

	Cases n=22	Control n=11	P–value
Age (years)	9.18 ± 2.80	8.90 ± 3.28	NS
Height (cm)	137.89 ± 17.06	130.64 ± 20.00	NS
Height (SDS)	0.87 ± 1.12	-0.009 ± 1.20	NS
Weight (kg)	63.30 ± 25.60	28.82 ± 11.62	P≤0.001
Weight (SDS)	5.85 ± 2.16	-0.37 ± 0.56	P≤0.001
BMI (kg/m^2^)	31.81 ± 6.93	16.31 ± 1.66	P≤0.001
BMI (SDS)	3.60 ± 0.63	-0.09 ± 0.89	P≤0.001
Waist Circumference (cm)	94.09 ± 15.33	56.64 ± 7.39	P≤0.0001
Hip Circumference (cm)	99.14 ± 17.44	65.00 ± 7.99	P≤0.0001
Waist/Hip Ratio	0.95 ± 0.073	0.87 ± 0.04	P≤0.001
SBP (mmHg)	119.55 ± 11.90	105.91 ± 9.44	P≤0.01
DBP (mmHg)	77.05 ± 10.66	65.45 ± 7.57	P≤0.01

SBP, systolic blood pressure; DBP, diastolic blood pressure; NS, non significant.

Vaspin concentration was higher in obese children than in controls. Similar difference was also elicited for serum visfatin. There was no significant difference in fasting blood glucose between the two studied groups, while significant elevation of serum insulin and insulin resistance (HOMA/IR) was observed in obese children relative to controls. Total cholesterol and LDL values were elevated in obese cases; no significant difference was detected in triglycerides and HDL in both groups under study (Table 2).

**Table 2:** Laboratory Data of Obese and Control Children.

	Obese children n=22	Control children n=11	P–value
Fasting glucose (mg/dl)	93.36 ± 10.43	91.45 ± 7.43	NS
Fasting insulin (mU/L)	17.3 ± 5.61	9.68 ± 5.70	P≤0.01
HOMA / IR	4.06 ± 1.51	2.11 ± 1.07	P≤0.01
Total Cholesterol (mg/dl)	194.55 ± 42.14	162.36 ± 17.92	P≤0.05
Total Triglycerides (mg/dl)	79.55 ± 34.21	67.45 ± 32.94	NS
HDL-C (mg/dl)	62.05 ± 20.80	73.18 ± 22.96	NS
LDL-C (mg/dl)	115.91 ± 40.18	75.82 ± 23.29	P≤0.01
Vaspin (ng/ml)	0.68 ± 0.25	0.33 ± 0.07	P≤0.01
Visfatin (ng/ml)	9.18 ± 3.04	4.33 ± 3.01	P≤0.05

HMA/IR = Homeostatic model assessment for insulin resistance; HDL-C=High density lipoprotein-cholesterol; LDL-C=Low density lipoprotein-cholesterol.

No correlations was found between serum vaspin and different demographic, laboratory and clinical studied data in obese children, except for a positive correlation between vaspin and waist hip ratio (P< 0.01, r = 0.7020). Table 3 shows the correlations with visfatin in obese children. Significant positive correlations were detected between visfatin level and height, weight, BMI, waist circumference, hip circumference and HOMA / IR of obese children.

**Table 3:** Correlations of visfatin level with demographic, clinical, and laboratory data in obese children.

	Serum visfatin level
Height	0.4878*
Ht-SDS	-0.051
Weight	0.5873**
Wt-SDS	-0.0922
BMI	0.4912*
BMI-SDS	0.2508
Waist circumference	0.6140**
Hip circumference	0.5775*
W/H ratio	-0.0333
Total cholesterol	-0,0308
Total triglycerides	0,1418
High Density Lipoprotein–cholesterol	0,0580
Low Density Lipoprotein–cholesterol	-0,0802
HOMA / IR	0,6046**
Systolic Blood Pressure	0,3739
Diastolic Blood Pressure	0,3626

* Spearman’s correlation is significant at the level 0.05;; means (P < 0.05).

** means (P < 0.01).

## Discussion

Obesity is one of the most serious risk factors for chronic diseases. It plays a central role in insulin resistance and metabolic syndrome [13]. Obese children in our study showed significant elevated serum insulin and insulin resistance (HOMA/IR) than controls. Obesity in childhood and adolescence is also associated with established risk factors for cardiovascular diseases and accelerated atherosclerotic processes [14]. Total cholesterol and LDL levels were elevated in obese cases in this study. Accelerated atherosclerotic processes are associated with elevated triglyceride and lower HDL [15]. We detected both changes in obese children but levels failed to reach significances in comparison to control children. Systolic and diastolic blood pressures were significantly higher in obese children. Same changes in blood pressure were reported by several authors [15, 16].

Cekmez et al in 2011 concluded that large for gestational age children had a higher vaspin and visfatin levels than those who are appropriate for gestational age [17]. Our data in obese children showed elevated levels of both vaspin and visfatin. Many studies concluded the same elevation of visfatin in obese children [18, 19]. Pagno et al., in 2006, found that plasma visfatin and its mRNA were significantly lower in obese subjects, compared with normal-weight controls [20]. Variation in results between studies may be related to genetic variations [21].

Administration of vaspin to obese mice improves glucose tolerance, insulin sensitivity and reduces food intake [22]. Vaspin may have antiatherogenic effects through its potential insulin-sensitizing properties and through its beneficial effects on the asymmetric dimethylarginine – endothelial nitrous oxide system [23]. It also protects vascular endothelial cells against free fatty acid-induced apoptosis through a phosphatidylinositol 3-kinase/Akt pathway [24]. In our study we did not find correlations between vaspin and cardiovascular risk factors, except with waist / hip circumference, which is not a reliable indicator for abdominal fat in children as waist cirumference [25]. Failure to detect such correlations may be related to different ages of cases in different studies and probably a small sample size.

Visfatin correlated with waist circumference and insulin resistance (HOMA/IR) in our study. Such correlation with Insulin resistance was elicited by Araki et al., in 2008 [26]. Insulin resistance expressed in HOMA/IR is more significantly interrelated with the metabolic syndrome components [27]. Waist circumference, a proxy measure of abdominal obesity, is associated with cardio-metabolic risk factors in childhood and adolescence [27]. Similar to atherosclerosis, abdominal fat is also one of the predictor risk factors of morbidity in obese individuals [28]. Adipokines may further contribute to obesity-atherosclerosis relationships, the full understanding of which will require much more researches [29].

In conclusion vaspin and visfatin levels increased in obese children. Visfatin related positively to abdominal fat and insulin resistance in the form of HOMA/IR; abdominal fat and insulin resistance are important indicators of metabolic syndrome in children. Vaspin is not a sensitive indicator of abdominal obesity and insulin resistance as visfatin. Still more researches are needed to explore its role in improving insulin tolerance.
